# Landmark Optimization Using Local Curvature for Point-Based Nonlinear Rodent Brain Image Registration

**DOI:** 10.1155/2012/635207

**Published:** 2011-09-26

**Authors:** Yutong Liu, Balasrinivasa R. Sajja, Mariano G. Uberti, Howard E. Gendelman, Tammy Kielian, Michael D. Boska

**Affiliations:** ^1^Department of Radiology, University of Nebraska Medical Center, 981045 Nebraska Medical Center, Omaha, NE 68198, USA; ^2^Department of Pharmacology and Experimental Neuroscience and Center for Neurodegenerative Disorders, University of Nebraska Medical Center, Omaha, NE 68198, USA; ^3^Department of Pathology and Microbiology, University of Nebraska Medical Center, Omaha, NE 68198, USA

## Abstract

*Purpose*. To develop a technique to automate landmark selection for point-based interpolating transformations for nonlinear medical image registration. *Materials and Methods*. Interpolating transformations were calculated from homologous point landmarks on the source (image to be transformed) and target (reference image). Point landmarks are placed at regular intervals on contours of anatomical features, and their positions are optimized along the contour surface by a function composed of curvature similarity and displacements of the homologous landmarks. The method was evaluated in two cases (*n* = 5 each). In one, MRI was registered to histological sections; in the second, geometric distortions in EPI MRI were corrected. Normalized mutual information and target registration error were calculated to compare the registration accuracy of the automatically and manually generated landmarks. *Results*. Statistical analyses demonstrated significant improvement (*P* < 0.05) in registration accuracy by landmark optimization in most data sets and trends towards improvement (*P* < 0.1) in others as compared to manual landmark selection.

## 1. Introduction

In rodent brains images may become distorted due to instrument imperfections or, in the case of histology, tissue processing. For example, magnetic field inhomogeneity causes geometric distortions in echo planar imaging (EPI) MRI; mechanical forces acting on a harvested brain during slicing may cause tissue tearing. And chemical preparation for histological analysis may cause deformation of tissue, evident in histological micrographs. Correcting (or “rectifying,” “warping”) the distorted images is required to adequately represent rodent brains (in the case of EPI distortion in MRI), or to compare different acquisition modalities (e.g., comparison of in vivo images and histological micrographs). Normally, affine (linear + translation) transformation cannot reconcile severe distortions making nonlinear transformation necessary. 

Among the nonlinear transformation techniques, point-based interpolating transformation techniques are widely employed because they are easy to implement and flexible for different applications [1–3]. A typical point-based interpolating approach is comprised of three steps: (1) placing homologous point landmarks on the source image (image to be transformed) and the target image (image used as the reference), respectively, (2) computing the interpolating transformation (e.g., polynomial splines, B splines and thin-plate splines [4, 5]) between the source and target images, and (3) aligning the landmarks exactly and mapping other parts of image using the computed transformation.

Accurate interpolating transformation requires an exact match of homologous landmarks. Manual identification of landmark points is time consuming and prone to intra- and interobserver variations. A number of investigators have attempted to automate the landmark definition process by exploiting the geometry of anatomical or biological structures. Typical geometrical features include line intersections [6, 7], local curvature maxima [8–10], and centroid of closed boundary region [11]. Employing the anatomical and biological features has greatly simplified the landmark generation. However, some biomedical images do not contain well-distributed features to generate reliable landmarks for accurate registration. More importantly, noise, artifacts, and other factors can cause errors when identifying the features using either automated or manual selection. A few studies have taken landmark location errors into account. Rohr et al. developed a method to relax the exact landmark matching (i.e., allowing the algorithm to relocate the landmarks) using thin-plate splines [12] by minimizing the bending energy functional [13, 14]. This method can cope with isotropic as well as anisotropic landmark errors. Bookstein [15, 16] used a linear regression model and a technique called “curve décolletage” to relax the interpolation condition. Image properties such as edges of objects [13] have been used to relax the exact landmark matching. However, the effect of the landmark relaxation on the registration accuracy was unknown in prior studies. 

In the current investigation, we developed a technique to automate and optimize the landmark generation using the local curvature on anatomical contours and validated the technique. The technique presented here is for two dimensional (2-D) brain image nonlinear registration. Although the rodent brain is a three dimensional (3-D) object, some brain imaging studies are essentially carried out on 2-D planes such as in histological microscopy, and in 2-D MRI studies. More importantly, nonlinear distortion mostly occurs on 2-D as well in these imaging studies. For example, tissue tearing, shearing, shrinkage, and enlargement, during sectioning and section handling, and eddy current in 2-D EPI MRI acquisition, mostly cause 2-D in-plane nonlinear distortions. To guarantee the correspondence of the two brain images to be registered, some prior process 3-D registration may be necessary. Detailed description of the process follows.

## 2. Materials and Methods

### 2.1. Algorithm Development

The strategy of this method is first to generate contours on corresponding anatomical features on the source and target images and then generate landmarks on homologous anatomical contours. The landmarks are then relaxed from the original locations and allowed to slide along the contours to achieve optimal matching. The relocation of the landmarks is governed by a cost function constituted by the local curvatures of landmarks and their displacements. The procedure is described using the pseudocode in Table 1.

The homologous contours on which the landmarks are generated can be manually drawn on the images or identified as the borders of objects using border detection methods, or first identified automatically using border detection methods and then manually modified to correct errors resulting from noise and artifacts. Depending on the image properties, appropriate border detection methods such as dynamic programming or active contour models [17] can be used. The homologous contours are manually split into several corresponding source and target curves that are relatively regular in shape (c.f. Figures 2(a), 2(b), 3(a), and 3(b). The splitting usually improves the computational stability and efficiency of the nonlinear transformation in our experience (we used the thin-plate splines for the transformation calculations).

**Figure 1:**
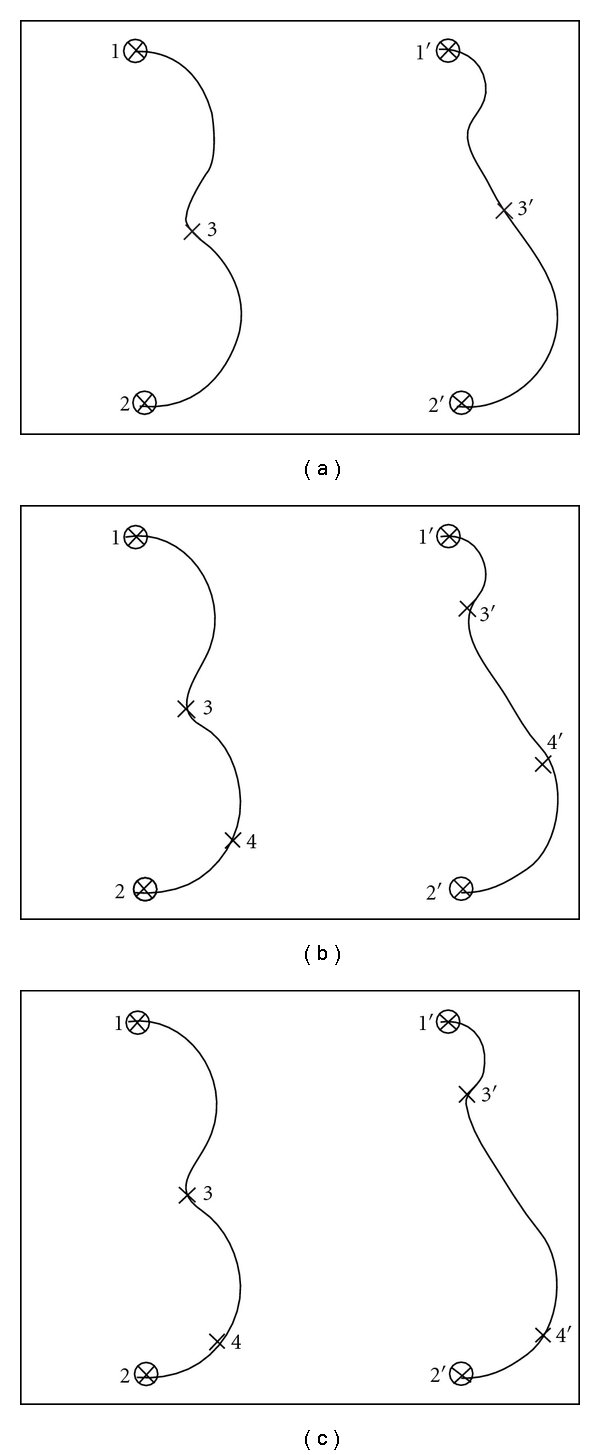
An example of the landmark generation and optimization. The left and right columns show the source and target curves, respectively. (a) The first two landmarks (1 and 2, circled X) and their homologues (1′ and 2′, circled X) are fixed at the two ends of the corresponding source and target curves. The 3th landmark (3) is placed in the middle of the longest segment of the source curve, and its homologue (3′) is placed on the target curve. (b) The landmarks (3 and 3′) are then relaxed from their initial locations and slid to match each other; and the 4th landmark (4) is placed in the middle of the longest segment of the source curve, and its homologue (4′) is placed on the target curve. (c) The 4th landmarks (4 and 4′) are then relaxed from their initial locations to match each other.

The next step is landmark generation and optimization on each curve. An example of this step is illustrated in Figure 1. The first two landmarks and their homologues are fixed at the two ends of the corresponding source and target curves (Figure 1(a)). The remaining landmarks (from the 3rd to the *n*th; *n* is the user-preset number of landmarks on the curve) are generated and optimized in an iterative fashion as shown in Table 1. The landmarks split the source and target curves into homologous curve segments (e.g., *i *− 1 landmarks split the curve into *i *− 2 segments). The *i*th landmark is placed in the middle of the longest segment of the source curve (can be on the target curve depending on the user; the source curve was used in this study), and its homologue is placed on the corresponding target curve segment (Figure 1(b)). Before the (*i* + 1)th landmark is added, the landmarks are relaxed from their initial locations and slid on the curve segments to match each other (this procedure is called landmark optimization, Figures 1(b) and 1(c)) by minimizing the cost function

(1) $$M=\left|\frac{\kappa_{i}^{S}}{\max\;\;\left(\kappa^{S}\right)}-\frac{\kappa_{i}^{T}}{\max\;\;\left(\kappa^{T}\right)}\right|+\lambda \cdot \left(\frac{\Delta_{i-1}^{S}}{l^{S}}+\frac{\Delta_{i-1}^{T}}{l^{T}}\right),$$

where *S* and *T* indicate the source and target, respectively, Δ_*i*−1_ is the displacement of landmarks on the (*i* − 1)th curve segment, *l* is the length of the curve segment, and *κ* is the local curvature defined by

(2) $$\kappa =\frac{||\dot{\gamma }\times \ddot{\gamma }||}{{||\dot{\gamma }||}^{3}},$$

where *γ* is a curve, “|| ||” represents the Euclidean distance, and “^·^” and “^··^” are the first and second derivatives, respectively. The first term of the cost function is the difference between the local curvatures (normalized by the maximum curvatures of the curve segments) of a source landmark and the homologous target landmark. The second term is the displacements of the landmarks from their initial locations normalized by segment length. The cost function is a combination of the curvature similarity of the source and target landmarks and the displacements of the landmarks. The landmarks are relaxed and moved to match their curvatures (the first term in (1)) to minimize the cost function, and their displacements are weighed by the second term. The weighting parameter **λ** > 0 regulates the sliding distances of the landmarks along the contours. By increasing **λ**, landmark displacement is restricted. Equation (1) is minimized using the Nelder-Mead algorithm [18]. When the landmarks have been optimized, any point-based registration method can be used to warp the source image and register the source and target images. We utilized the thin-plate splines [12] to generate the warping field for registering the target images to the source in this work.

### 2.2. Algorithm Evaluation

The landmark generation and optimization technique was evaluated using two types of mouse brain imaging studies. In the first study, mouse brain MRI was nonlinearly registered with histological sections. The second study examined the ability of the algorithm to correct geometric distortion of EPI—a fast MRI acquisition technique. The convergence criterion of the cost function minimization was set as either landmark displacement of less than 10^−4^ pixels in subsequent iterations or a maximum of 500 iterations, and the weighting factor **λ** was set to between 0.2 and 0.3 in both of these studies. The thin-plate splines, which have been extensively used in medical image registration [7, 19–30], were used for the nonlinear transformations. The accuracy of registration between the transformed source image (MRI in the first study, and EPI in the second study) and the reference image was measured by two methods using the normalized mutual information (NMI) and target registration error (TRE), respectively. NMI is defined as

(3) $$\text{NMI}=\frac{\left(H\left(S_{t}\right)+H\left(T\right)\right)}{H\left(S_{t},T\right)},$$

where *H*(*S*_*t*_) and *H*(*T*) are the marginal entropies, respectively, of the transformed source image *S*_*t*_ and *T*, and *H*(*S*_*t*_, *T*) denotes their joint entropy. TRE [3] is the distance between a set of target points (**P**_*T*_) and warped corresponding source points (**P**_*S*_). In general,

(4) $$\text{TRE}=T\left(P_{S}\right)-P_{T},$$

where *T* is the transformation, which is the thin-plate splines in this study. Therefore TRE is an array of vectors. In this study, only the average magnitude of TRE is of interest and thus reported. The size of the point set was dependent on the nature of the images, and at least four points were identified on each pair of images. 

#### 2.2.1. Registration of MRI to Histological Sections

Mouse brains were harvested after 3D T2*-weighted MRI, fixed and embedded in paraffin for histological sectioning. The blockface of the embedded brain was photographed during sectioning (blockface imaging). Individual blockface images were stacked to reconstruct the 3D brain volume. Brain slices were stained with Prussian blue and hematoxylin. The MRI volume was linearly registered to the blockface volume, and then computationally resliced in the coronal plane to match the corresponding histological sections [31, 32]. The MRI slices and histological sections were then nonlinearly registered by three experienced technicians using the thin-plate splines with the landmarks generated and optimized by the presented technique. The technicians were instructed to draw the source and target contours and generate curves according to their knowledge of anatomy. The automatic landmark optimization technique was evaluated by comparing two registration results: the first was from landmarks generated on the user defined contours but adjusted manually by the technicians, and the other registration was conducted using discrete landmarks manually selected by the technicians.

#### 2.2.2. Correction of EPI Distortion

Five mice were scanned with T_2_-weighted (T_2_-wt) spin-echo imaging and diffusion tensor imaging (DTI) with EPI acquisition. The MRI slices were prescribed at the same anatomical locations in these scans. In the DTI EPI scan, a baseline image without diffusion weighting (B0) was also acquired. The B0 images were used as the source images, and the T2-wt images were used as the target images for registration. Since the B0 image and all diffusion-weighted images undergo same geometric distortion, the transformation procured from the T2-wt/B0 registration is applied to correct the diffusion-weighted images. The B0 images were registered to the T2-wt images by the same three technicians in the MRI/histology registration using manually selected landmarks, landmarks manually adjusted on anatomical contours, and automatically generated and optimized on contours, respectively.

## 3. Results

### 3.1. Registration of MRI to Histological Sections

**Figure 2:**
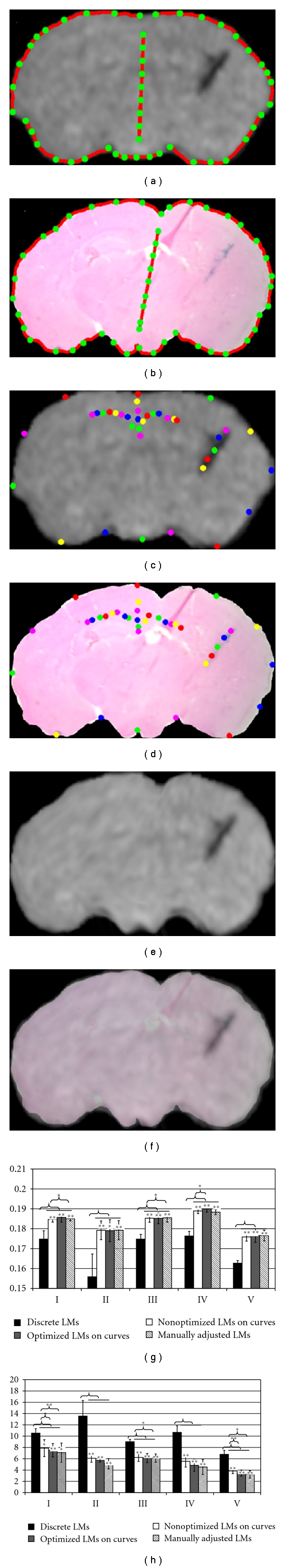
The registration of MRI and histological slices using optimized landmarks on curves (a) and (b), manually selected discrete landmarks (c) and (d). The colors of homologous landmarks are matched in (c) and (d). The MRI slice transformed using the thin-plate splines with optimized landmarks in (a) and (b) is shown in (e), and overlaid on the histological section (f). The average NMI and TRE of transformed MRI and histological slices in five image pairs (I–V) were shown in (g) and (h), respectively. The NMI and TRE were calculated on the MRI slices transformed using manually selected “discrete landmarks (LMs)” (black columns), landmarks generated on curves but not optimized (“Nonoptimized LMs on curves”) (white columns), curve landmarks optimized using (1) (“Optimized LMs on curves”) (gray columns), and curve landmarks manually refined (“Manually adjusted LMs”) (stripped columns) *: *P *< 0.1; **: *P* < 0.05.

Figures 2(a) and 2(b) demonstrate a pair of MRI and histological slices before coregistration. The curves drawn by a technician and landmarks generated and optimized on the curves are also shown on these figures. The transformed MRI and its overlay on the histological section are shown on Figures 2(e) and 2(f). Figures 2(c) and 2(d) show the discrete manually selected landmarks by a technician. The landmarks that were manually adjusted on curves are not shown.

Registration results with landmarks generated using different methods in five pairs of MRI and histological slices (from Pair I to V) were compared. The mean and standard error of the NMI and TRE of each pair of images by the three technicians is presented in Figures 2(g) and 2(h), respectively. NMI and TRE are shown from the manually selected discrete landmarks (“discrete landmarks (LMs)” in Figures 2(g) and 2(h)), landmarks generated on anatomical curves and optimized using (1) (“Optimized LMs on curves” in Figures 2(g) and 2(h)). The NMI and TRE of landmarks on curves but not optimized (“Nonoptimized LMs on curves”) and of landmarks manually adjusted on curves (“Manually adjusted LMs”) are also presented to investigate the improvement in registration accuracy resulting from landmark optimization. The number of optimized, manually adjusted and nonoptimized landmarks, was the same on each curve. The nonoptimized landmarks were obtained by simply generating the landmarks on the curves without performing optimization using (1), keeping each landmark at its initial position. 

As demonstrated in Figure 2(g) (NMI results) and Figure 2(h) (TRE results), generating landmarks on anatomical curves (no matter the landmarks were optimized or not) either significantly improved registration accuracy (*P* < 0.05, paired *t*-test) or showed a trend towards improvement (0.05 ≤ *P* < 0.10) in all image pairs compared to manually selecting discreet landmarks. The automatic landmark optimization using (1) resulted in a trend towards registration accuracy improvement (0.05 ≤ *P* < 0.10) in one image pair by NMI calculations (Figure 2(g)) compared to nonoptimized landmarks, and a trend towards improvement (0.05 ≤ *P* < 0.10) in two image pairs by TRE calculations (Figure 2(h)). Compared to nonoptimized landmarks, manually adjusted landmarks showed a trend toward improvement in registration accuracy (0.05 ≤ *P* < 0.10) on two image pairs by NMI (Figure 2(g)). The TRE results showed a significant accuracy improvement (*P* < 0.05) or a trend towards improvement (0.05 ≤ *P* < 0.10) on three image pairs by manually adjusting the landmarks (Figure 2(h)). No difference was found between automatically optimized and manually adjusted landmarks.

### 3.2. Correction of EPI Distortion

**Figure 3:**
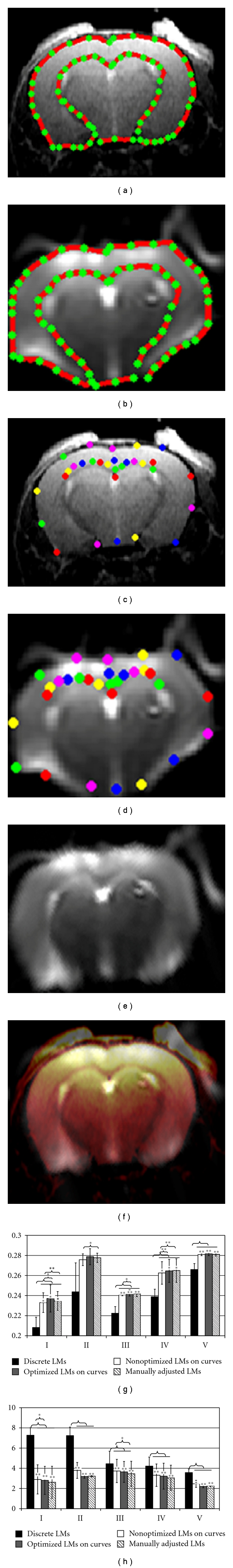
The correction of the geometrical distortion on a B0 image (a) in EPI DTI employing the T2-wt image (b) as the reference by optimized landmarks on curves (a) and (b), and manually selected discrete landmarks (c) and (d). The colors of homologous landmarks are matched in (c) and (d). The B0 image transformed using the thin-plate splines with optimized landmarks in (a) and (b) is shown in (e), and overlaid on the T2-wt image plotted in pseudocolor (f). The average NMI and TRE of corrected B0 and T2-wt images in five image pairs (I–V) were shown in (g) and (h), respectively. NMI and TRE were calculated on the B0 images transformed using manually selected “discrete landmarks (LMs)” (black columns), landmarks generated on curves but not optimized (“Nonoptimized LMs on curves”) (white columns), curve landmarks optimized using (1) (“Optimized LMs on curves”) (gray columns), and curve landmarks manually refined (“Manually adjusted LMs”) (stripped columns) *: *P* < 0.1; **: *P* < 0.05.

A typical pair of B0 and T2-wt images is shown in Figures 3(a) and 3(b). The geometrical distortion is evident on the B0 by visual comparison to the T2-wt images. Figures 3(a) and 3(b) show the anatomical curves and optimized landmarks on a pair of T2-wt/B0 images, and the discrete landmarks manually selected by the same technician are shown in Figures 3(c) and 3(d). The result of distortion correction using the optimized landmarks is shown in Figure 3(e). For an improved visualization, the corrected B0 (in grayscale) is overlaid on the T2-wt image plotted in pseudocolor in Figure 3(f).

The same statistical analysis was performed in the EPI distortion correction as for the MRI/histology registration, allowing a direct comparison of the results (Figures 3(g) and 3(h)). Generating landmarks on anatomical curves (irrespective of whether or not the landmarks were optimized) either significantly improved registration accuracy (*P *< 0.05) or showed a trend towards improvement (0.05 ≤ *P* < 0.10) in all image pairs compared to manually selecting discreet landmarks except in one image pair (in Figure 3(g), by NMI calculations). The automatic landmark optimization using (1) resulted in significant accuracy improvement (*P* < 0.05) in one image pair and a trend towards registration accuracy improvement (0.05 ≤ *P* < 0.10) in two image pairs by NMI calculations (Figure 3(g)) compared to nonoptimized landmarks, and a trend towards improvement (0.05 ≤ *P* < 0.10) in one image pair by TRE calculations (Figure 3(h)). Compared to nonoptimized landmarks, manually adjusted landmarks showed a significant improvement (*P* < 0.05) in registration accuracy on two image pairs by NMI, and a trend toward improvement (0.05 ≤ *P* < 0.10) on one image pair (Figure 3(g)). The TRE results showed a trend towards improvement (0.05 ≤ *P* < 0.10) on one image pair by manually adjusting the landmarks (Figure 3(h)). No difference was found between automatically optimized and manually adjusted landmarks.

## 4. Discussion

In this study, we developed a landmark generation and optimization technique for point-based nonlinear image registration methods. This technique extracts landmarks from the anatomical contours and optimizes the landmark positions by minimizing the cost function constituted by the displacements and the local curvatures of the landmarks. The technique was evaluated in two distinct applications: the registration of MRI and histological slices and distortion correction of EPI MRI images. Statistical analyses have shown that the automation of landmark selection resulted in significant accuracy improvement in image registration compared to manually selected landmarks. Although in most experiments the improvement in NMI and TRE resulting from the landmark optimization was not statistically significant compared to the results using nonoptimized landmarks, the trends towards improvement in registration accuracy was demonstrated in several experiments. Manually adjusting the landmarks on curves could improve registration accuracy on several experiments and show a trend towards improvement in some other experiments. We found no difference in registration accuracy between landmark automatic optimization and manual adjustment. However, automated landmark selection provides increased efficiency by minimizing the required user intervention. 

We used two methods including NMI to validate our technique. NMI has been intensively used as an image similarity measure. It is not a monotonic function of the image similarity and, thus, may be trapped at local minima when used as a driving force for image registration. In this study, the image pairs to compare have all been already registered; thus, NMI was only calculated on a small interval on which it is reasonable to think that NMI is monotonic. TRE was also calculated for the registration evaluation in this study. TRE is a measure of the registration accuracy of a set of points on the images. The points for TRE calculation are usually identified on anatomical features; thereby, the evaluation may be more meaningful than NMI with regard to the anatomical accuracy of registration.

The anatomical contours were manually drawn in this study. Border detection methods can be used to automatically identify the contours. It is reasonable to think that automatically generating contours can minimize inter- and intrauser variance. But on the other hand, some automatic border detection techniques are more susceptible to noise and artifacts compared to manual delineation. We are currently investigating the registration accuracy using different border detection methods. 

Not only can this method be used in imaging studies similar to those presented here, but also can be used for multiple brain registration, or similarly, for registration to an atlas with minimum modification. Before using this method, a 3-D affine transformation is likely necessary to first align the brain volumes together, or to the atlas, and then each brain volume needs to be resliced to match individual brain slices, or to the atlas slices.

Overall, this method results in improved registration accuracy and efficiency. However, this technique still requires user intervention and thus suffers inter- and intra-investigator inconsistencies. We are currently improving this technique by including the image intensity and more geometrical information in addition to displacement and curvature to fully automate the landmark generation process. In this study, the number of landmarks either manually selected or automatically generated on curves was determined by the technicians according to their experience. It is desirable to precalculate the necessary landmark number for different landmark generation methods to improve registration accuracy. Investigations using previously published methods to accomplish this automation are underway [33].

## Figures and Tables

**Table 1:** Pseudocode of the landmark generation and optimization technique.

(1)Identify homologous contours on the source and target images either by manually drawing or using image processing techniques.
(2)Split the homologous contours into *C* pairs of corresponding source and target curves.
(3)For *c* = 1 to *C *
For *i* = 3 to *n *
Add the *i*th landmark on the middle of the longest segment of the *c*th source curve and the homologous landmark on the corresponding target segment.
Optimize landmarks by minimizing the cost function in (1).
End
(4)End
